# Climate and Diseases of New Mexico

**Published:** 1865-02

**Authors:** 


### CHICAGO MEDICAL EXAMINER.

N. S. DAVIS, M.D., Editor.

VOL. VI. FEBUARY, 1865. NO. 2.

### Original Contributions.

ARTICLE IV.

### CLIMATE AND DISEASES OF NEW MEXICO. REMARKABLE EXEMPTION FROM CONSUMPTION. PREVALENCE OF RHEUMATISM AND SYPHILIS.

The statement has been made by a French writer, that tubercular consumption does not originate in regions elevated 5000 feet or more above the sea. Knowing that the Territory of New Mexico had an elevation varying from 4000 to 10,000 feet, and that it had shown a remarkable freedom from tubercular disease, according to the statistics of the U. S. Army, I felt strongly desirous of ascertaining whether it might not be the best place to which we could send consumptively inclined patients from this region. I, therefore, addressed a letter of inquiry to Dr. O. M. Bryan, Surg. U.S. Vols., and Med. Director at Santa Fé, N. Mexico. I present his reply to the readers of the Examiner, in the following pages. Surgeon Bryan is known to the profession of this State as the former Secretary of the Illnois State Board of Medical Examiners.

E. ANDREWS, M.D.

Santa Fe, N. M., Nov. 10, 1864.

Doctor Andrews—*Dear Sir:*—In reply to your letter, making inquiries in regard to the character of diseases peculiar to this country, the effect of high latitudes upon diathesis, etc.,

I would say: that your questions can be more correctly and satisfactorily answered by furnishing you, from the Hospital records, the necessary statistics.

I, therefore, enclose herewith a consolidated meteorological register for the year 1863, taken from the records of the hospital at Fort Marcy, Santa Fé, N. M. Also, a consolidated report of sick and wounded for the same year.

From the former, you will be able to obtain much important information in regard to the peculiarities of the climate of Santa Fé; and from the latter, you can form some idea of the varieties of disease we have to treat in this department; you cannot, however, from this report obtain any information in regard to the diseases peculiar to Santa Fé, from the fact, patients are brought here for treatment, from all parts of the Department, from an altitude of near 3500 to that of near 7000 feet. You will observe from this report, that there were 32 cases of intermittent fever treated during the year; in my opinion, not one of them originated in Santa Fé. The 269 cases treated during the year, in hospital at Fort Marcy, are classified as follows:—

| | |
|---|---|
| Fevers, continued, 1; typhoid, 1; intermittent quot. 32,- | 34 |
| Diseases of the organs connected with the digestive system, | 25 |
| Diseases of the respiratory system, | 40 |
| Diseases of the brain and nervous system, | 5 |
| Diseases of the genital organs and venereal affections, | 74 |
| Diseases of the fibrous and muscular structure, | 12 |
| Abscesses and Ulcers, | 6 |
| Wounds and Injuries, | 49 |
| Diseases of the eye, | 4 |
| Diseases of the ear, | 3 |
| All other diseases, | 17 |

Out of the entire number, there were only six deaths: one from delirium tremens; one from syphilis consec'd; one from anasarca; one from debility; and two from pneumonia. I would here remark, that the 269 cases referred to, were composed of troops from California as well as from New Mexico.

For the past eighteen months, I have been on duty in Santa Fé; and during that time have had a good opportunity of observing the character of diseases common to the natives of the city and county about. Agreeable to your request, I will give you the names of the diseases most frequently met with among the native population of this section of New Mexico, and to the best of my judgment the ratio per 100 of each:—

| | | | |
|---|---|---|---|
| Gonorrhœa, | 15 | Pneumonia, | 3 |
| Syphilis, | 8 | Paralysis, | 4 |
| Rheumatism, | 10 | Chorea, | 1 |
| Dyspepsia, | 10 | Scrofula, | 6 |
| Diarrhœa, | 6 | Erysipelas, | 3 |
| Dysentery, | 3 | Anasarca, | 4 |
| Hepatitis, | 3 | Ascites, | 2 |
| Catarrh, | 12 | Typhoid Fever, | 2 |
| Bronchites, chronic, | 3 | Conjunctivitis, | 5 |

You will be surprised at the large percentage of venereal disease; but if you could live in this country, and become acquainted with the manners and customs of the people, you would find the Mexican population in a state of complete moral degradation, unsurpassed, in my opinion, on the face of the earth. The natives (with a few exceptions) consider it no more of a disgrace to be suffering from a venereal disease, than from a bad cold; and both sexes will converse with you freely upon the subject without a blush. I will relate you a case that occurred in my practice a few days ago, that will give you some idea of the morals of this country. A Mexican, of good family and some little wealth, called at my office, and requested me to visit his wife and daughter, who were living some three miles distant from the city, and were both suffering severely from syphilis. I promised to do so next morning. Found the wife a woman of about 35 years of age, and, upon examination, found a large syphilitic chancre, which she informed me was about *two weeks old;* told me she contracted the disease from her husband, and that the husband contracted his from a prostitute in Santa Fé. The husband acknowledged the statement correct. As far as I could judge, there was a perfect good feeling between the parties, and they seemed to look upon the matter simply as a *bad accident.*

I next examined the daughter, a girl about 15 years of age; found her in bed, perfectly helpless, with syphilitic rheumatism; had been confined to her bed over two months; was very much emaciated; soft palate nearly gone from ulceration, together with the power of articulation; bones of the nose were carious, &c. She informed me that she contracted the disease from a soldier, who kept her as his mistress, some months ago.

Cases similar to the one I have related are by no means uncommon in New Mexico.

Rheumatism, in all its forms, is another disease peculiar to this country. I am satisfied that altitude has but little to do with producing or aggravating the disease, from the fact that about the same proportion of cases are reported to me monthly from the different posts between here and Franklin, Texas; and we find a difference in altitude between the two points of over 3000 feet. All classes—the rich and the poor—the American with all his comforts and luxuries, as well as the poor Mexican, who is obliged to suffer severe exposures, at the same time destitute of proper food and clothing, are liable to suffer more or less from rheumatism.

You will observe, from my list of diseases, that dyspepsia is one of the *ranking* diseases of the country. All classes suffer from it, and, in my opinion, it is produced, to a great extent, from the enormous amount of chile (red pepper) that they consume. They eat it three times per day, if they can get it, sometimes cooked alone and sometimes with meat. If you ask a sick Mexican if he is suffering much pain, the common reply is, "Se, Senor; mi estomago nee duele mucho." Yes, sir; I have much pain in the stomach.

Colds and coughs are also very common here in the winter season. They are produced by living in warm and crowded rooms, poorly ventilated, and exposure to the cold air without sufficient clothing. Another cause of the constant hacking cough that the Mexicans are so subject to, is, the habit both sexes have of constantly smoking their cigaritas. They are generally made of tobacco rolled in *corn husks;* the smoke of the husks has a tendency to irritate the air-passages and produce a cough. Colds and coughs seldom result in anything serious, although I have seen, in Santa Fé, cases both of bronchitis and pneumonia result fatally.

We often see, in this altitude, cases of scrofula among the lower classes, evidently produced by exposure, bad diet, uncleanliness, living in crowded and badly ventilated rooms, &c.

Since I have been on duty in Santa Fé, I have had to treat among the natives five cases of hemiplegia (all between 50 and 60 years of age)—one case of pariplegia, that proved fatal—one case of malignant erysipelas, that attacked the upper lip and proved fatal in a few days. Cases of simple erysipelas are by no means uncommon. Have operated upon four natives (all middle age) for paracentisis abdominis; three out of the four have since died; the fourth, I believe, will recover. Have met with two cases of typhoid fever during the past 18 months.

I have never seen a case of tetanus, cancer, typhus fever, or malarious disease of any kind that I had reason to believe originated here. We occasionally have epidemics of scarlet fever, measles, small-pox, and whooping-cough among the children, that is truly frightful and fearfully fatal. The cause of the great mortality among children is very evident; poorly clad, poorly fed, and poorly lodged. There is snow on the ground at the present moment, but if you could walk through the streets of Santa Fé, you might see, perhaps, 50 children nearly naked, and many literally so.

You will observe that I have not included tuberculosis among the diseases common to this country. From obsrrvation, I am satisfied that tuberculosis should never originate in New Mexico; but when you take into consideration the manner in which the lower classes live, in total violation of every hygeinic law, it is really surprising that half the population are not suffering from scrofula and pulmonary tuberculosis. Since I have been here, I have had the opportunity of witnessing and making a careful examination in two cases of *genuine* tuberculosis that originated and terminated fatally in Santa Fé. Both cases were women, one 23 and the other 45 years of age; the former was born and raised in this city, and the latter was born in old Mexico, but contracted the disease in Santa Fé. I mention these cases to show that tuberculosis *does originate* at an altitude of near 7000 feet.

You ask if consumptives removing to Santa Fé obtain decided benefit. If the disease is far advanced, I think not; if in the first stage, I think they do. In my opinion, there are places in this Department far superior to Santa Fé for pulmonary disease. My observation teaches me that the altitude here is too great, the atmosphere too rarefied, and the winters too cold for consumptives.

For over three years past I have been suffering from chronic bronchitis. I will now give you my experience in regard to the different localities in this Department, which I find agrees with the experience of others with whom I have conversed suffering with pulmonary disease.

After having a hard trip across the Plains, riding day and night and coughing most of the time, I arrived in Santa Fé Nov. 12th, 1862 (altitude 6846 feet). My breathing, as I approached the city and after arriving here, became very much labored, my respiration hurried, great oppression about the chest, and a sensation that I should have hemorrhage of the lungs if I made much physical exertion. These symptoms were so distressing, that I requested Surgeon E. J. Bailey, U.S.A., then Medical Director of the Department, to order me immediately down the river. After remaining in Santa Fé three days, I was ordered to Los Perios, a military post situated on the Rio Grande, 79 miles south of Santa Fé (altitude 4932 feet). All the unpleasant symptoms above described began to disappear as I descended the river, and left me entirely while at Los Perios; could see but little improvement in my cough.

About the middle of January, 1863, I was ordered to La Mesilla, N. M.; a town located on the Rio Grande, 280 miles below Santa Fé (altitude 4000 feet). Here my breathing became natural, and my cough began to improve—(there is, however, too much wind and dust at La Mesilla and Las Cruces to make them desirable points for a consumptive). About the 1st of March I was ordered to Franklin, Texas; a delightful military post on the Rio Grande, just opposite the City of El Passo, in old Mexico; 326 miles south of Santa Fé, and 46 miles south of La Mesilla (altitude about 3700 feet).

I consider Franklin by far the most desirable post in the Department for a consumptive. It is so situated (partially surrounded by mountains) that you escape, to a great extent, the severe winds and dust that you find at most other points on the river.

My health here improved very rapidly, and in less than three months I gained 8lbs. in flesh. The mercury at this post seldom falls below 50 or rises above 90. On the 14th of May, I received an order from the Secretary of War to report for duty to Gen. CARLETON, at Santa Fé. I arrived here on the 24th of the same month, and have been on duty here from that date. For months after my arrival here from Franklin, I suffered, to some extent, the same symptoms as described above; oppression about the chest, hurried respiration, &c.; my cough slightly increased. Last winter I endeavored to run a few rods from my office to the Post Office; was attacked with hemorrhage from the lungs, that confined me to my room for several days. Since then my health has gradually improved; am now quite strong and cough but little; am of the opinion that if I could have remained in Franklin for one year, my health would have been completely restored.

If any of your consumptive patients intend visiting this country for their health, advise them to make Franklin their *head quarters*. If the disease is not too far advanced, it might be of benefit to spend the summer months in Santa Fé, as our summers are cool and delightful. After receiving your letter of Aug. 30th, I wrote to Assistant-Surgeon JOHN BROOKE, U.S.A., on duty at Albuquerque, N. M., also to Assistant-Surgeon A. H. SMITH, U.S.A., on duty at Las Cruces, N. M., to send me reports and statistics from their posts on the points you required; I enclose them herewith for your information. I would here state that Albuquerque is 63 miles south of Santa Fé, altitude 5032 feet, and Las Cruces 277 miles south of Santa Fé, altitude 3970 feet.

Should have answered your letter long ago, but have been waiting for the reports from Drs. Brooke and Smith.

Yours very respectfully,

O. M. BRYAN.

---

Las Cruces, Oct. 17th, 1864.

Doctor:—It gives me great pleasure to comply, so far as I can, with your request of the 7th inst.

I regret to say that there was no meteorological register kept at this post during the year 1863. When I arrived here there was not even a thermometer in the hospital, and I have just received, for the first time, a hygrometer. I have had no register, except an old one which had been used by the Texans to keep a mess account.

The diseases most prevalent here are as follows, the percentage of each being stated from my impressions as to their relative frequency, not among the troops alone, but among the citizens also:—

| | | | |
|---|---|---|---|
| Gonorrhœa, | 20 | Rheumatism, | 10 |
| Syphilis, | 8 | Miasmat. Fevers, | 8 |
| Diarrhœa, | 20 | Bronchitis, | 3 |
| Dysentery, | 8 | Pneumonia, | 1 |
| Conjunctivitis, | 5 | | |

There are two forms of miasmatic disease prevailing in this locality. The most frequent is a variety of intermittent, usually quotidian in the beginning, but becoming tertian under the influence of quinine, to which it yields completely in the course of six or eight days. It is very rare that all the phenomena of a "chill" are present, the first and third stages being often omitted, and the second stage represented by neuralgia or cephalalgia. Indeed, in many cases the periodicity is the only characteristic which points to a miasmatic origin. The other form is a mild type of remittent fever, showing a strong disposition to terminate in a number of days divisible by seven, and rarely exceeding twenty-one. This form of fever made its appearance about two months ago, and is probably owing to the large amount of rain which has fallen this year, and which has left a great deal of stagnant water in the vicinity. It is much more prevalent among the citizens than among the troops. Of typhus fever I have not seen a single case; also, not one of simple typhoid.

Genuine pulmonary tuberculosis, I think, *never* originates in this section. I have seen two or three cases of hemorrhage from the lungs, but without any of the physical signs of tubercles. The natives speak of a rare disease which they call *consumcion*, but they locate it in the bowels. From the description, I judge that the term is applied to the wasting away, from chronic disease, of some of the chylo-poietic viscera.

The effect of the climate upon tuberculosis originating elsewhere is eminently favorable. During the eleven months that I have been here, several cases have come under my observation, all of which have been benefited in a marked degree. This season has been remarkably wet, and, therefore, comparatively very unfavorable, otherwise still greater benefit might have been derived. I am inclined to ascribe the advantages of the climate in this respect to the extreme dryness of the atmosphere, rather than to the altitude. The average fall of rain at Fort Fillmore, eight miles from here, is nine inches and a fraction, being less than at any other military post in the United States, Soccon excepted. I have enquired of Dr. Samaniego, of El Paso, with regard to tuberculosis in his locality, and he stated that he had seen only two cases among the natives during a practice of several years. Doubtless the result of protracted observation here would be similar.

I have seen several cases of scrofula among the natives, and both acute and chronic hydrocephalus are not uncommon. The mode of life of the lower class of the natives, which is in defiance of every hygienic law, undoubtedly conduces greatly to this result.

Rheumatism is exceedingly prevalent, both the acute and chronic forms. I think this is owing, at least in part, to the cellar-like character of the adobe houses, and the practice of keeping the floors constantly damp. Troops in tents are not more affected, I think, than they would be in the States.

I have seen but one case of erysipelas since I have been here, and that was idiopathic. The readiness with which wounds heal has passed into a proverb among the laity. I have not seen or heard of a single case of tetanus. I have now in hospital a case of melanotic cancer, the only one which has come under my observation.

In the absence of meteorological observations for this post, I enclose a copy of some notes of observations at Fort Fillmore, taken some time since from the Army M. Register. The average mean temperature for each month for a period of five years, 1851–5, is given:—

| | | | | | |
|---|---|---|---|---|---|
| Jan., | 44.47. | May, | 71.32. | Sept., | 77.21. |
| Feb., | 48.93. | June, | 80.92. | Oct., | 64.39. |
| March, | 55.96. | July, | 83.36. | Nov., | 51.22. |
| April, | 64.39. | Aug., | 79.68. | Dec., | 46.45. |

Lat. 32° 13′. Lon. 106° 42′. Altitude, 3937 feet.

This letter was furnished me by Assistant-Surgeon A. H. Smith, U.S.A., on duty at Las Cruces, for the past 13 months.

O. M. BRYAN.

Albuquerque, N. M., October 31, 1864.

The diseases which prevail in this locality are those which are common to most parts of the world, irrespective of climate or situation. So far as my observations have extended, there are no positive endemics. Syphilis, more especially the numerous forms it takes in the secondary and tertiary stages, is probably the most common disease. Rheumatism is very frequent. Pneumonia, bronchitis, dysentery, and diarrhœa, chronic disease of the liver, and the usual eruptive fevers, constitute the larger proportion of the remaining diseases.

Of typhus or typhoid fevers, tetanus, or cancer, I have never seen a case, and but few of erysipelas. The only cases of malarious fevers which have come under my notice were in persons who had had previous attacks; in fact, simply relapses brought on by some exciting cause. The effect of the climate on rheumatism is, I think, unfavorable, especially near and among the mountains, a few miles distant from the river. Genuine scrofula is exceedingly rare, if not entirely unknown. Those cases having the appearances of scrofula generally occur in persons whose systems are tainted with the poison of syphilis, either acquired by the individuals themselves, or inherited from their parents.

The influence of the climate on tubercular consumption is, I should think, highly beneficial. I judge so from negative evidence, for I have seen but one well-marked case in this place, and that was in a soldier with whom the disease had made such extensive progress before he came to this country, that any other than a fatal result was physically impossible. I have never seen a case occurring among the natives of the country; although the manner in which the large majority of them live—improper and insufficient food, want of cleanliness, exposure to cold, and imperfect ventilation—is highly favorable to the production of tubercular disease. With the predisposing and exciting causes so prevalent, the almost total absence of it can only be accounted for by the elevation, and the peculiar salubrity of the climate.

The mortality among the native population of this country is fearfully large; and, looking at climatic influences alone, perfectly unaccountable. I have never seen statistics, but I should judge from observation that the population if at all on the increase, is very slightly so indeed. There have been no wars largely destructive of human life, and no emigration from the country; and yet the growth of the towns and villages is so slow as to be almost imperceptible, while there are but few of them that cannot boast of more than a century's existence.

A large majority of native children are small and puny at birth, and greatly deficient in *vital force.* This is attributable to two causes. The first is, the criminal practice of intermarriage between blood relations, which exists to a frightful extent, and produces the usual result, of imperfectly developed offspring. This custom is permitted by law, and *sanctioned* by the Church. The second cause is transmitted syphilitic taint. A large number of these children die at birth, or within a few days after. With the exception of a fortunate few, those who survive have insufficient food; their clothing—*if clothed at all*—is alike in the hottest weather of summer and the coldest of winter; through the day they run, unwashed and wild, through all manner of dirt and filth; and sleep, when the nights are cold, in rooms densely crowded, and an atmosphere fouled by smoke, filth, and a careful prevention of all ventilation. With constitutions thus weakened and robbed of their vitality, it is no wonder that measles, hooping-cough, and scarlatina—diseases which, under the most favorable circumstances, are so often fatal to children—as well as the usual ailments attendant upon dentition, should find hundreds and thousands of easy victims. The puny abortions of humanity are swept away in frightful numbers by these diseases, and whole families are desolated by a single epidemic. If they have the stamina to resist the depressing influences by which they are surrounded in early life, and pass the age of puberty with vitality but little impaired, they nearly always pass into hale and hearty manhood. In fact, a New Mexican who carries a good constitution into middle life, stands a fair chance of seeing fourscore, or even a hundred years. They pass a "green" old age, and reach a *dry* one; and, if Sam. Weller's theory concerning the disappearance of postboys and donkeys be applicable to Mexicans and burros, it is not unreasonable to suppose that these desiccated specimens of mortality "venever they feels themselves getting stiff and past their work, just rides off in the usual way, to take their pleasure in some other vorld."

Could the present inhabitants of this country be entirely swept away, leaving behind them no traces of their superstitions, their follies, or their crimes, and the land peopled with a race who were uncontaminated by hereditary disease, and who lived according to the simplest teachings of nature, the result would be a state of physical perfection and longevity unknown to the modern world.

The above document has been furnished me by Assistant-Surgeon John Brooke, U.S.A., on duty at Albuquerque, for the past 22 months. O. M. BRYAN.

